# An accurate deep learning model for wheezing in children using real world data

**DOI:** 10.1038/s41598-022-25953-1

**Published:** 2022-12-28

**Authors:** Beom Joon Kim, Baek Seung Kim, Jeong Hyeon Mun, Changwon Lim, Kyunghoon Kim

**Affiliations:** 1 Department of Pediatrics, College of Medicine, The Catholic University of Korea, Seoul, Republic of Korea; 2 Department of Applied Statistics, Chung-Ang University, 84 Heukseok-Ro, Dongjak-Gu, Seoul, 06974 Republic of Korea; 3 Department of Pediatrics, Seoul National University Bundang Hospital, Seongnam, 13620 Republic of Korea; 4 Department of Pediatrics, Seoul National University College of Medicine, Seoul, Republic of Korea

**Keywords:** Health care, Medical research, Mathematics and computing

## Abstract

Auscultation is an important diagnostic method for lung diseases. However, it is a subjective modality and requires a high degree of expertise. To overcome this constraint, artificial intelligence models are being developed. However, these models require performance improvements and do not reflect the actual clinical situation. We aimed to develop an improved deep-learning model learning to detect wheezing in children, based on data from real clinical practice. In this prospective study, pediatric pulmonologists recorded and verified respiratory sounds in 76 pediatric patients who visited a university hospital in South Korea. In addition, structured data, such as sex, age, and auscultation location, were collected. Using our dataset, we implemented an optimal model by transforming it based on the convolutional neural network model. Finally, we proposed a model using a 34-layer residual network with the convolutional block attention module for audio data and multilayer perceptron layers for tabular data. The proposed model had an accuracy of 91.2%, area under the curve of 89.1%, precision of 94.4%, recall of 81%, and F1-score of 87.2%. The deep-learning model proposed had a high accuracy for detecting wheeze sounds. This high-performance model will be helpful for the accurate diagnosis of respiratory diseases in actual clinical practice.

## Introduction

Auscultation of the lung is the oldest and most widely utilized method for respiratory examination and is useful for the diagnosis and follow-up of various lung diseases^1^. For example, wheezing is a common sign in children with acute episode of asthma, and rale is helpful in the diagnosis of pneumonia^1,2^. The stethoscope is an inexpensive, non-invasive, non-radioactive, easy to perform tool that requires minimal time for diagnosis^3^. Therefore, it remains an essential tool, although methods for evaluating the respiratory system are rapidly developing. However, it has obvious limitations, such as subjectivity, requiring extensive experience and training, inability to save and share, and impossible to continuously monitor^4^. It can lead to inaccurate diagnosis that can adversely affect patient care.

Deep learning, an artificial neural network-based technology, is an artificial intelligence (AI) tool that solves complex problems by learning on its own from raw data, without human intervention^5,6^. When deep learning is applied in the medical field, more objective and accurate results are provided, and time and resources can be efficiently used because there is no need to manually extract features^6,7^. Recently, automatic analysis using deep learning has been actively applied in various medical fields, such as medical imaging, medical informatics, bioinformatics, and pervasive sensing, and excellent effects have been reported^5^. For example, a meta-analysis reported that the accuracy of automatic image analysis was similar to that of an expert, and there was a report that the research speed and results of drug development and genomic analysis also improved^5,7^. In addition, the development of wearable devices is actively progressing^5^.

Recently, deep-learning models that classify normal breathing sounds and adventitious sounds have been actively studied^8,9^. These models achieved high agreement with classification by conventional auscultation. Several studies have reported that this model outperforms clinicians' auscultation. The performance of this AI model was comparable to that of a pulmonologist and showed superior accuracy compared with residents, general practitioners, and students^3,10,11^. However, there are not many studies verifying deep-learning models that classify breathing sounds from actual clinical situations, and there are many studies using open databases, which have limitations in reflecting actual clinical situations^12–14^. Additionally, there are few studies with pediatric patients, either too few subjects or too few specific types of abnormal sounds^3,12^. This limitation in the amount and quality of available data can estimate the performance and generalization of lung sound classification systems^15^.

We hypothesized that developing a model that automatically analyzes adventitious respiratory sounds by learning breathing sounds through deep learning may help overcome the limitations of using a stethoscope. However, to apply this existing model in clinical practice, it is necessary to develop a performance model that reflects accurate clinical information. The aim of this study was to construct an AI algorithm that could be applied in actual clinical practice in detection of wheezing, which is an adventitious respiratory sound, and perform data augmentation to compensate for the limitations in the amount of available data. Furthermore, we aimed to collect real-world patient information and high-quality breathing sounds. Finally, we tried to achieve optimal performance by applying residual network (ResNet) with convolutional block attention module (CBAM) techniques based on convolutional neural networks (CNN).

## Methods

### Study design and data collection

This prospective study included children who visited the Department of Pediatrics at university hospitals in Korea from August 2019 to January 2020. We recorded the breathing sounds of patients who voluntarily consented to recording breathing sounds. Recordings were performed in an outpatient clinic by a pediatric pulmonologist using an electronic stethoscope (Jabes, GSTechnology, Seoul, Korea). According to the diagnosis of the specialist, the auscultation sounds were recorded by classifying wheezing and other respiratory sounds. Four breathing sounds were obtained per patient by recording the anterior and posterior regions of both lungs for two cycles. To verify the classification, blinded validation was performed by two pediatric pulmonologists, and if one or more classifications were the same as the existing classification, they were tagged and stored in the database. Additionally, we collected data on sex, age, and location.

### Evaluation of AI algorithm

**Figure 1:**
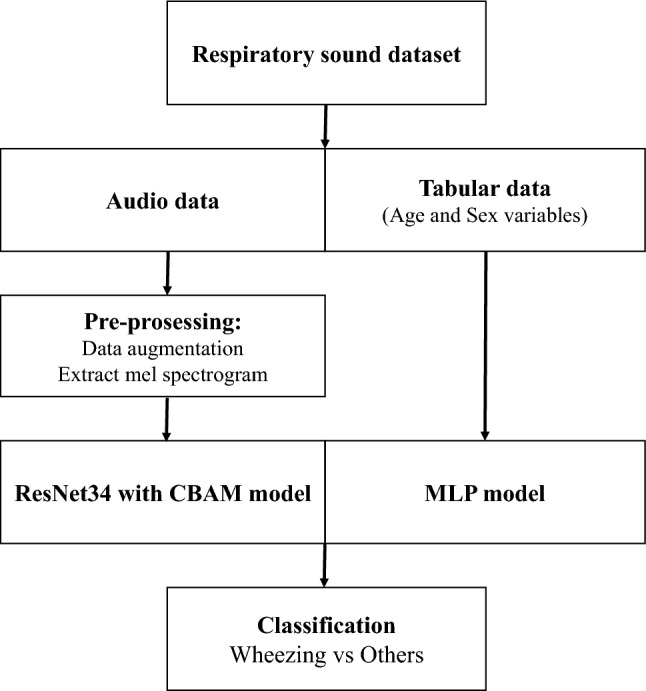
Flow chart of the classification of respiratory sound using deep-learning model. The mel spectrogram extracted from the audio data pass the ResNet34 with Convolutional Block Attention Module (CBAM) model, and tabular data pass through the Multilayer Perception (MLP) model.

**Figure 2:**
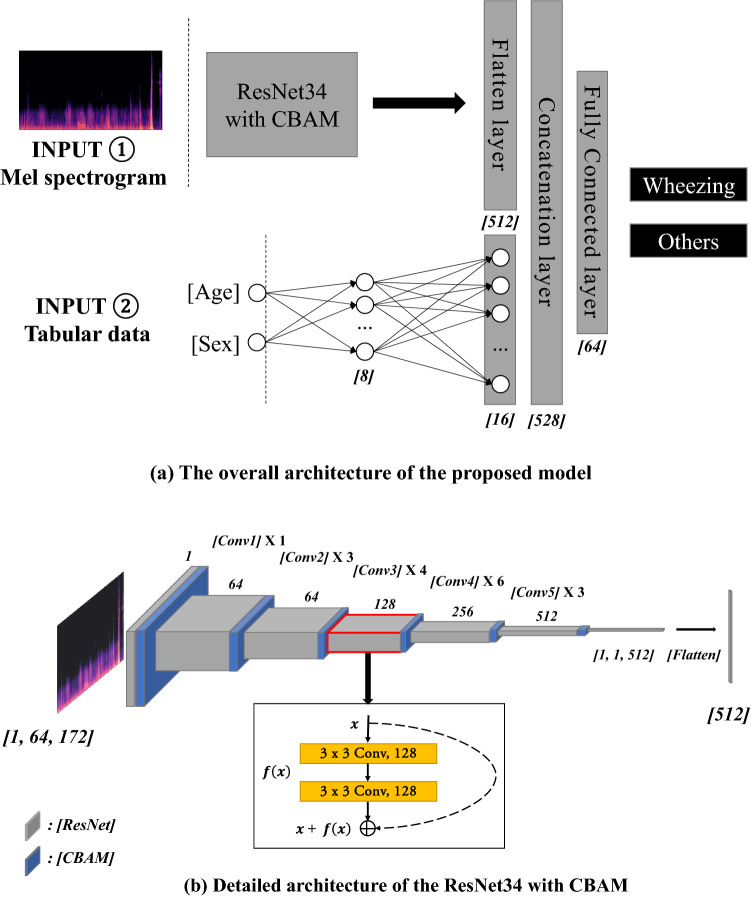
The architecture of the proposed model. (**a**) The model classifies respiratory sounds by connecting 512 features output from the ResNet34 with Convolutional Block Attention Module (CBAM) model with 16 features output from the Multilayer Perception (MLP) model. (**b**) [1, 64, 172] size input data convolved with a 3 × 3 size filter. Finally, extraction to 512 size.

We constructed a binary classification model to determine whether breathing sounds contained wheezing sounds. We used 80% of the database as training data and 20% as test data. The mel spectrograms extracted from audio data through the pre-processing process, gender labeled with 0 (female) and 1 (male), and normalized age were used as input data. We propose a deep-learning model that consists of ResNet34 with CBAM for audio data and multilayer perceptron (MLP) layers for tabular data (Fig. 1). We confirm that tabular data improves performance through the 4-layer CNN with tabular data model, and finally apply the ResNet34 with CBAM and tabular data model^16,17^. A mel spectrogram from the breathing sound of size [1, 64, 172] was used as the input data. Initially, a 7 × 7 convolution filter was used, and then a 3 × 3 convolution filter was used. A total of 512 features were output, and gender and age data passed through the MLP model consisting of 8 and 16 nodes, respectively. Finally, it becomes a model to determine whether wheezing occurs through a fully connected layer (Fig. 2). Our experiments used Python version 3.6.5. The entire process of training and testing the proposed model is presented in a block diagram and presented as a supplementary figure (Supplement A). The ResNet and CBAM structures used to deal with breathing sounds in our proposed model are as follows:

### ResNet

CNN with deep layers extract high-level features. However, in a layer that is too deep, the performance decreases^18^. Therefore, we used ResNet with skip connection techniques to solve these problems and 34-layer models^19^.

### CBAM

There have been many attempts to apply an attention mechanism to improve performance based on the CNN structure. The attention mechanism is based on the idea that when humans see objects, they do not process the whole object at once and focus on the visible part. The attention mechanism shows where to focus on the model using weights. In the CNN architecture, various attempts have been made to apply an attrition mechanism in the form of a module to improve the performance of the model^20–22^. CBAM is a lightweight attention module that leads to significant performance improvement, even with very little overhead. It consists of two types of attention map: channel attraction and spatial attraction.

This model focused on improving performance by efficiently training the weight pattern of respiratory sounds through the attention mechanism, and also focused on lighting by adopting a relatively light model. Additionally, tabular data were configured to help train through the MLP layer.

(A) Pre-Processing
  1. Data augmentation: We augmented the training data by applying the following six augmentation techniques: white noise addition, time shifting, stretching, reverse, minus, and fit transform (Supplement B). The librosa package was used to augment 230 pieces of training data into 1610 pieces of learning data and extract a mel spectrogram^23^.
  2. Feature Extraction: Mel-spectrograms were extracted from the audio data of breathing sounds. A 1024-point fast Fourier transform processor and a 64-bit mel filter bank was implemented in this process. We performed repeat padding for a variable length of sound data. Shorter samples were repeated from batch to batch with the maximum sample size. The torchaudio package was used in a previous work^24^. In addition, time masking and frequency masking of the specifications were performed to avoid overfitting the learning data to the model. Finally, the mel spectrogram was normalized and used as the input data.

(B) Optimal construction and validation of AI model
  We used the cross-entropy loss function and Adam optimizer for the deep learning. To find the optimal hyper-parameters, five-fold cross-validation and the grid search method were used^25^ (Supplement C). The proposed model was learned over 120 epochs, and the batch size was 32, and learning rate was 0.0001. The hyper-parameters of all the models, including those compared, are presented in Table 2. We applied the stochastic weight average technique, which updates the weight's average value every cycle to further boost the performance^26^. The performance of the model was evaluated using a test dataset. We obtained accuracy, precision, recall, F1-score, and area under the curve (AUC) values. We compared the performance with the following models: a 3-layer long short-term memory (LSTM) model, a 4-layer CNN model, a model that adds tabular data to the 4-layer CNN model. We used a PyTorch framework that is compatible with torchaudio used in pre-processing to build a deep-learning process.
  Additionally, for comparison with models known in previous studies, VGG16, VGG19, InceptionV3, ResNet50, and ResNet101 pre-trained on ImageNet were compared with our models by validating their performance using respiratory sound data from this study^11^.

### Statistical analysis

To compare the characteristics of respiratory sounds, we used the chi-squared test (for discrete variables) and Mann–Whitney *U* test (for continuous variables). Analyses were performed using SPSS (version 22.0; SPSS Inc., Chicago, IL, USA), with the probability level for significance set at a *P* value of < 0.05.

### Ethics statement

This study was approved by the Institutional Review Board (IRB) of the Catholic University of Korea (IRB approval no. PC19OESI0045). Written informed consent was obtained from at least one legal guardian for all participants. For children 7 years of age and older, assent of child was also obtained. All methods were performed in accordance with relevant guidelines and regulations.

## Results

### The characteristics of the respiratory sound database

Seventy-six patients in the clinical field were enrolled in this study, and 103 wheeze sounds and 184 other respiratory sounds were stored in the dataset. Table 1 shows the characteristics of the respiratory sound database, such as sex, age, location of auscultation, and duration of sound.

**Table 1:** Characteristics of recordings used in the study.

	Wheezing (n = 103)	Others (n = 184)	*P* value
**Demographic data for recording subjects**			
Male, *n* (%)	67 (65.0)	113 (61.4)	0.541
Age (year)	4 (2–8)	3 (1–5)	< 0.001
**Location of auscultation, *n* (%)**			0.883
Anterior, left	23 (22.3)	46 (25.0)	
Anterior, right	29 (28.2)	49 (26.6)	
Posterior, left	28 (27.2)	44 (23.9)	
Posterior, right	23 (24.4)	45 (24.5)	
Duration of sound (ms)	89.36 ± 39.51	63.09 ± 27.79	< 0.001

Continuous variables are expressed as mean ± SD or median (IQR).

All the recorded files had a sample rate of 48,000 Hz. However, the Python library used for data augmentation supports a maximum sample rate of 22,050 Hz; therefore, we down sampled all files accordingly. Respiratory sounds have different lengths and were set equally to 273.61 millisecond through repeat padding.

### Performance of proposed model and comparison with other experiment models

Table 2 presents the performance comparison between the proposed model and other experimental models such as the 3-layer LSTM model, 4-layer CNN model, and 4-layer CNN model with tabular data. The 3-layer LSTM model for CNN and recursive neural networks (RNN) comparison showed an accuracy of 82.4%, AUC of 84.1%, precision of 70.3%, recall of 90.4%, and F1-score of 79.1%. The 4-layer CNN model was set as the basic model that detected wheeze sounds with an accuracy of 88.7%, AUC of 85.5%, precision of 87.4%, recall of 75.6%, and F1-score of 81.3%. When tabular data, including age and sex, were added to the basic model, the performance improved compared to that of the basic model. It had an accuracy of 89.5%, AUC of 87.7%, precision of 89.8%, recall of 80.5%, and F1-score of 85%.

**Table 2:** Performance for discriminating other respiratory sounds from wheezing.

Models	Selected hyper-parameters with grid search	Accuracy	AUC	Precision	Recall	F1-score
3-layers LSTM	Epoch: 40/batch size: 32/ Learning rate: 1e−3/dropout rate: 0.2	0.824	0.841	0.703	0.904	0.791
4-layers CNN	Epoch: 60/batch size: 16/ Learning rate: 1e−2/dropout rate: 0.4	0.887	0.855	0.874	0.756	0.813
4-layers CNN + Tabular data	Epoch: 60/batch size: 16/ Learning rate: 1e−3/dropout rate: 0.4	0.895	0.877	0.898	0.805	0.850
34-layers ResNet with CBAM + Tabular data	Epoch: 120/batch size: 32/learning RATE: 1e−3	0.912	0.891	0.944	0.810	0.872

AUC, area under the curve; CNN, convolutional neural network; CBAM, convolutional block attention module; LSTM, long short-term memory; ResNet, residual network.

The proposed model, a CNN-based 34-layer ResNet with a CBAM, and tabular data exhibited the highest performance among the experiments based on the CNN model. The model had an accuracy of 91.2%, AUC of 89.1%, precision of 94.4%, recall of 81% and F1-score of 87.2%.

Table 3 shows the performance of VGG16, VGG19, InceptionV3, ResNet50, and ResNet101 pre-trained on ImageNet, as reported in a previous study using respiratory sound data^11^.

**Table 3:** Comparison of performance of existing models pre-trained on ImageNet proposed in a previous study using our respiratory sound dataset. (Kim et al. 11).

Models	Accuracy	AUC	Precision	Recall	F1-score
InceptionV3	0.841	0.838	0.772	0.809	0.794
VGG16	0.820	0.831	0.764	0.795	0.781
VGG19	0.852	0.844	0.806	0.812	0.810
ResNet34	0.877	0.873	0.818	0.857	0.837
ResNet50	0.859	0.849	0.809	0.810	0.809
ResNet101	0.877	0.863	0.850	0.809	0.829

AUC, area under the curve; ResNet, residual network; VGG, visual geometry group network.

## Discussion

In this study, respiratory sounds were prospectively collected from pediatric patients in actual clinical practice. In addition, pediatric pulmonologists with abundant clinical experience carefully recorded the respiratory sounds and verified them by blind validation. Therefore, our dataset is comparable to any gold standard for deep learning because it reflects the real world, has a high sound description accuracy, and has a high sample rate. We developed a deep-learning AI model to classify wheezing using CBAM in a CNN-based ResNet structure. This model has a sufficiently high performance to be useful in actual clinical practice. We also found that adding tabular data to deep-learning models improved performance.

Recently, various methods have been proposed to improve the performance of deep-learning models of lung sound classification. The use of CNN, RNN, and other methods has been proposed as deep-learning architectures. Among them, several studies have evaluated CNN as most optimal for the respiratory sound classification model^27,28^. CNN operates the neural network by applying convolutional operations and is used in various fields such as image, video, and natural language interpretation. Recently, CNNs have also been frequently used in tasks using audio, and many models have been derived by transforming and upgrading CNN^29^.

The CNN model we adopted as a basic structure can extract abundant features and learn efficiently as the layer deepens^30^. However, overfitting may occur as the layer becomes deeper, which increases the complexity of the model and reduces performance^30^. Based on CNN, several hybrid models have been proposed to compensate for such problems and achieve optimal performance^15,28,31^. As for the most recent research, a model with performance higher than that of the existing breathing sound classification models by adding artificial noise addition technique to the general CNN structure has been proposed^28^. Moreover, a study proposed a model that achieved good performance using the combination of the co-tuning and stochastic normalization techniques of the CNN-based pre-trained ResNet as backbone^15^.

We tried to achieve optimal performance by applying ResNet with skip connection techniques based on CNN. ResNet is characterized by preventing overfitting and increasing performance using residual learning and skip connections^16^. In addition to ResNet, various feature extractors, such as the inception network (InceptionNet), dense network (DenseNet), and visual geometry group network (VGGNet) have been proposed to solve gradient loss and overfitting^32^. A recent study reported that VGG16 use pre-trained on ImageNet had the best performance in the detection of abnormal lung sounds, with an AUC 0.93 and an accuracy of 86.5%^11^. We tested the performance of respiratory sound classification by applying the same model as that tested in our previous study. As a result, the ResNet we adopted performed the best.

LSTM is a model of the RNN family, used for data with continuous properties^33^. Since respiratory sound data can be viewed as time series data with continuous properties, the LSTM model is also suitable for respiratory sound classification. Petmezas et al.^31^ used a hybrid CNN-LSTM structure and a local loss to solve the data imbalance. The lung sounds data were input for CNN, and the obtained output was used as an input for the LSTM. However, in general, it is known that CNN models learn features better than RNN models when learning audio data^34,35^. We confirmed that the performance of a typical LSTM family is lower than that of a typical CNN family through the performance comparison of the models.

We improved the performance by adding CBAM to CNN. An attention mechanism has recently been proposed to effectively deal with sequence problems^36^. The attention module uses weights to focus more on important parts and less on relatively unimportant parts^36^. In our study, the CBAM was introduced to improve the performance by giving weight to the mel spectrogram of the part where the wheeze pattern exists, and the accuracy improved by 1.7% compared to before the introduction. In addition, we constructed a multi-modal configuration to use not only respiratory sound data but also tabular data, such as age and gender information, for classification. We found that this model improved performance compared to the model using only breathing sound data. In particular, the increase in F1 scores was the most notable. It can be inferred that adding tabular data to the algorithm helps solve the problem of unbalanced data. Further research is required to confirm this hypothesis.

In previous studies, the deep-learning model was trained using only audio data without considering variables such as gender and age of the patient^11,12^. However, the characteristics of lung sound may differ slightly depending on gender and age, and to consider them together, a multi-modal model including tabular data of gender and age was constructed. In addition, a previous study, reported a model, combining tabular data with images, that solved the class imbalance between normal and abnormal data in the classification of chest radiographic images and reported improved image classification based on the sensitivity metric^37^. In our study, addition of the MLP layer showed an improvement in all performances, including the F-1 score, compared to CNN alone.

Several previous studies on CNN-based AI for lung sound classification used an open-access sound database, such as the International Conference on Biomedical and Health Informatics (ICBHI) 2017^12–14^. The ICBHI dataset contains a large number of respiratory sounds and events, including wheezes, crackles, cough, and noise^38^. However, such open-access data may have selection bias. In fact, some sounds from the ICBHI dataset are collected in non-clinical environments, some are from healthy patients, and some have not been double-validated^38^. In addition, there is a possibility that only certain sounds may be emphasized because of the short respiratory cycle of recordings^39^. In particular, when examining an actual patient, crying sounds and other breathing sounds may be auscultated. Therefore, research using open-access data is difficult to apply in the real world. The audio data used in this study were recorded in an actual clinical setting and verified by experts to increase accuracy. Therefore, our database is an excellent gold standard for constructing AI models that are useful in clinical practice.

This study had several limitations. First, this was a single-center study with a small sample size. We split our data and used 80% for training and 20% for validation. Furthermore, we used data augmentation and repeat padding to overcome the limitation in the amount of data for deep learning. Large amount of real-world data needs to be collected through a multicenter prospective study in the future. In addition, there was a problem with imbalanced data in the training dataset. We adopted the F1 score to solve the problem using metrics, and our model showed a high performance. Second, our model is a binary classification model that differentiates sounds that contain wheezing. For real-time monitoring, a deep-learning model needs to be developed through the advancement of data and AI performance that can be applied to various breathing sounds in the future. Finally, we did not collect patients’ diagnostic information. Diagnosis of lung disease is based on a comprehensive assessment of the patient’s clinical symptoms, laboratory test results, and breathing sounds. Development of a model for diagnosing diseases and evaluating the response to treatment by integrating this information is warranted through future studies.

## Conclusion

In this study, we propose a deep-learning model with a high accuracy for detecting wheeze sounds. CNN-based ResNet with CBAM as a classifier showed high performance in respiratory sound classification. Because this model requires a small amount of computation and memory space, it can be implanted on mobile devices; therefore, it is thought to be useful in actual clinical practice. In addition, we found that tabular data contributed to the performance improvement of deep-learning models for the classification of respiratory sounds.

## Supplementary Information

Supplementary Information 1.

Supplementary Information 2.

Supplementary Information 3.

Supplementary Information 4.

## Data Availability

The datasets generated and/or analyzed during the current study are available from the corresponding author upon reasonable request.

## Abbreviations

AI: Artificial intelligence
AUC: Area under the curve
CBAM: Convolutional block attention module
CNN: Convolutional neural network
DenseNet: Dense network
ICBHI: International Conference on Biomedical and Health Informatics
InceptionNet: Inception network
MLP: Multilayer perception
ResNet: Residual network
VGGNet: Visual geometry group network
