# Author Correction: An accurate deep learning model for wheezing in children using real world data

**DOI:** 10.1038/s41598-023-28376-8

**Published:** 2023-01-20

**Authors:** Beom Joon Kim, Baek Seung Kim, Jeong Hyeon Mun, Changwon Lim, Kyunghoon Kim

**Affiliations:** 1 Department of Pediatrics, College of Medicine, The Catholic University of Korea, Seoul, Republic of Korea; 2 Department of Applied Statistics, Chung-Ang University, 84 Heukseok‑Ro, Dongjak‑Gu, Seoul, 06974 Republic of Korea; 3 Department of Pediatrics, Seoul National University Bundang Hospital, Seongnam, 13620 Republic of Korea; 4 Department of Pediatrics, Seoul National University College of Medicine, Seoul, Republic of Korea

Correction to: *Scientific Reports* https://doi.org/10.1038/s41598-022-25953-1, published online 28 December 2022

The original version of this Article contained an error in the spelling of the author Kyunghoon Kim which was incorrectly given as Kyung Hoon Kim.

Additionally, the Article contained an error in the Author Information section.

“These authors contributed equally: Beom Joon Kim, Baek Seung Kim, Chang Won Lim and Kyung Hoon Kim.”

now reads:

“These authors contributed equally: Beom Joon Kim and Baek Seung Kim.

These authors jointly supervised this work: Chang Won Lim and Kyunghoon Kim.”

The original Article has been corrected.

