# Supplementary material for: An accurate deep learning model for wheezing in children using real world data
Source: Sci Rep. 2022 Dec 28;12:22465. doi: 10.1038/s41598-022-25953-1 (PMC9797543; doi:10.1038/s41598-022-25953-1)
Supplement: Supplementary file 1 — Supplementary Information 1. [file 41598_2022_25953_MOESM1_ESM.docx]

# Additional files:

## Additional file A. Block Diagram of the Proposed Model Architecture

## Additional file B. Visualization Data Augmentation Method

(a) Add white noise, (b) Time shifting, (c) Time stretching, (d) Reverse, (e) Minus, (f) Fitch transform

## Additional file C. Five-Fold Cross Validation
