# Supplementary figures and images for: An accurate deep learning model for wheezing in children using real world data
Source: Sci Rep. 2022 Dec 28;12:22465. doi: 10.1038/s41598-022-25953-1 (PMC9797543; doi:10.1038/s41598-022-25953-1)

## Slide 1
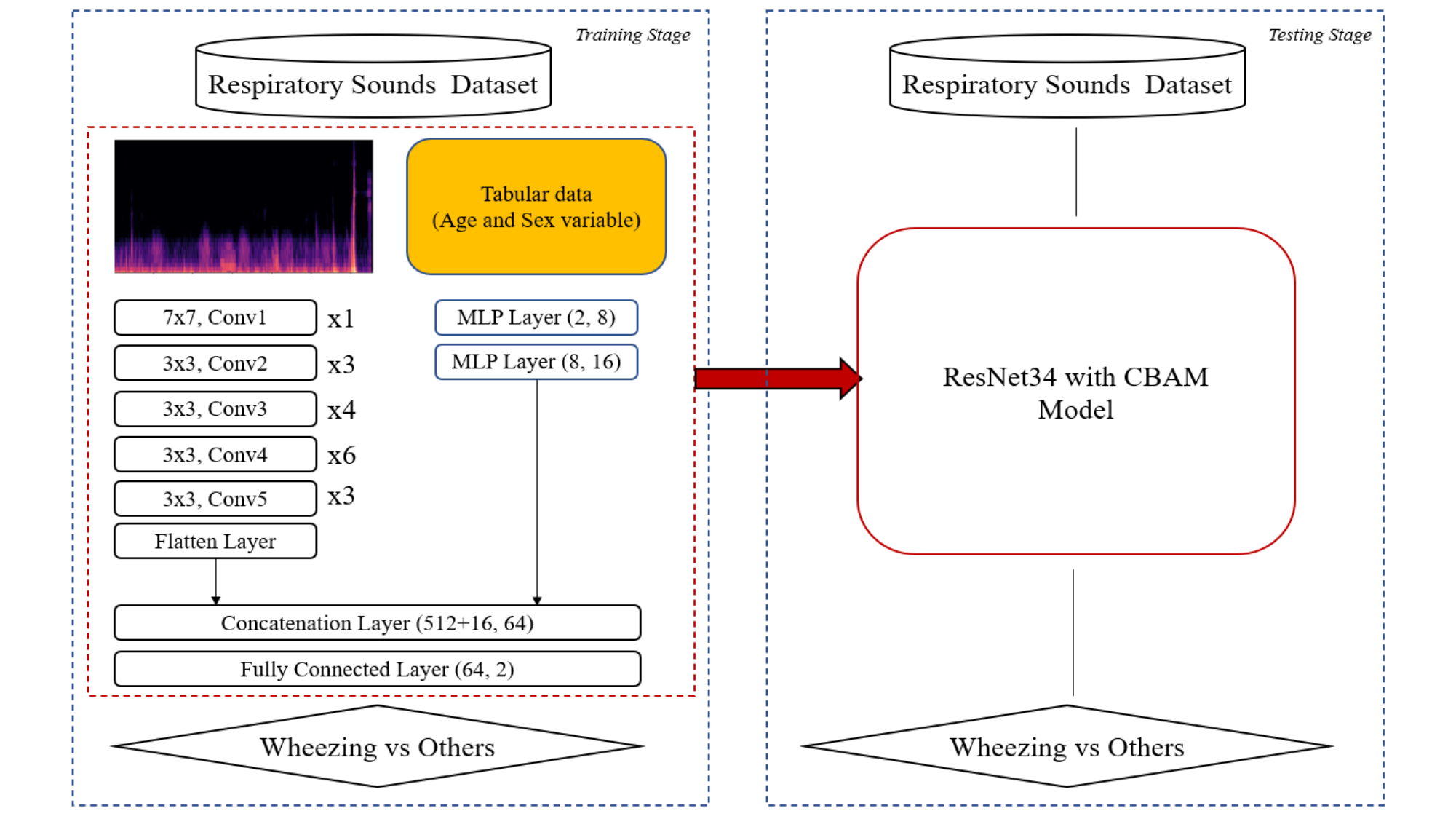

Supplement: Supplementary file 2 — Supplementary Information 2. [file 41598_2022_25953_MOESM2_ESM.pptx]

## Slide 1
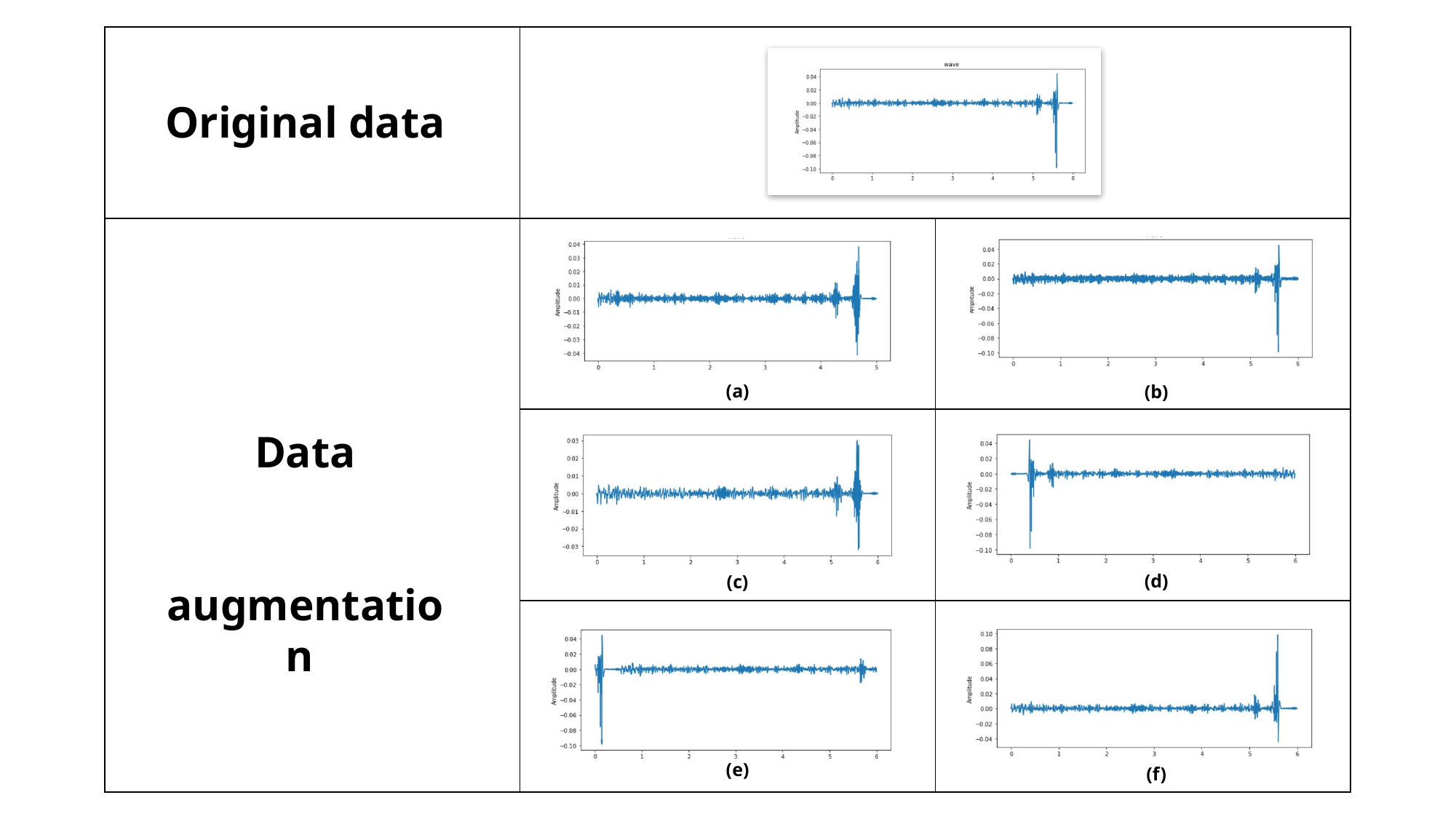

| | | |
| --- | --- | --- |
| | | |
| | | |
| | | |
Original data
(a)
(b)
Data
 augmentation
(d)
(c)
(e)
(f)

Supplement: Supplementary file 3 — Supplementary Information 3. [file 41598_2022_25953_MOESM3_ESM.pptx]

## Slide 1
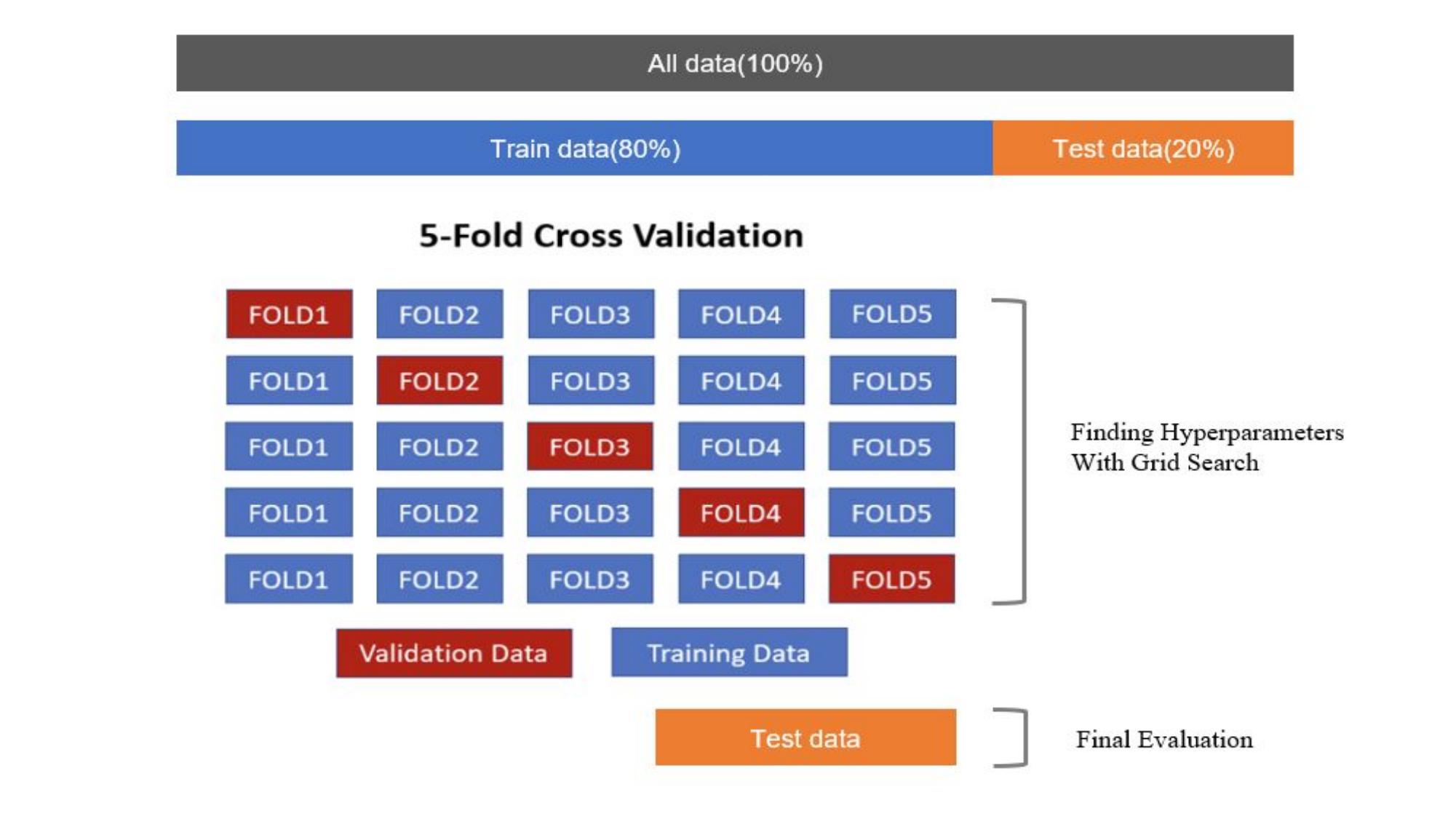

Supplement: Supplementary file 4 — Supplementary Information 4. [file 41598_2022_25953_MOESM4_ESM.pptx]
